# A System of Gynæcology

**Published:** 1897-12

**Authors:** 


**A System of Gynæcology.** By Many Writers. Edited by THOMAS CLIFFORD ALLBUTT, M.D., F.R.S., and W. S. PLAYFAIR, M.D. Pp. xviii., 972. London: Macmillan and Co., Ltd. 1896.

This system is a most welcome contribution to the literature of the subject, and the editors are to be congratulated on the completion of their task. To look over the list of its eminent contributors is sufficient to show that the book is stamped with the authority of some of the best British workers in this branch of medicine and surgery. The collaboration of "many writers" has great advantages, inasmuch as the editors are enabled to choose men specially suited for the different sections. There are, however, a few drawbacks, as in the overlapping of allied subjects. Moreover there is a tendency for each man to treat his assigned portion in an academic manner, so that manipulative details, which we are apt to assume wrongfully to be matters of common knowledge, are omitted. The book is not nominally divided into chapters in the ordinary way; and this departure is not a happy one, as reference is made rather more difficult.

After giving a full and clear account of the development of gynæcology, the book proceeds to the anatomy of the female pelvic organs; their malformations; and the etiology and diagnosis of disorders connected with them. In the section on inflammation of the uterus Dr. Barbour rightly insists on the importance of constitutional treatment, and maintains that far too much importance has been given to local medication. As an example of moderation in local treatment, we notice that the author would limit curettage for endometritis—which has undoubtedly been much abused—"to cases in which there is a clear history of recent abortion, in which there is considerable menorrhagia which has not yielded to ergotine, or in which the sound shows the cavity to be distinctly enlarged and roughened with vegetations."

Those of us who have not been impressed by Apostoli's method of the treatment of uterine fibroids, will read with approval the temperate article of Dr. Milne Murray on the electrical treatment of diseases of women. In a concise and masterly account of extra-uterine fœtation Mr. Bland Sutton maintains that it cannot be urged with too much force that "as soon as it is fairly evident that a woman has a tubal pregnancy, it should be dealt with by operation without delay,"—a conclusion which is supported by the greatly improved results of operation in this condition, and by the fact that "extra-uterine children" are generally ill-developed and rarely live long.

In the article on hysterectomy it is interesting to find that Mr. Knowsley Thornton says that "the results completely bear out the impressions I had formed, or rather support still more strongly the extraperitoneal method, with Koeberlé's serre-nœud for the ordinary run of cases"—an important contribution to the rival advocacy of the intra- and extra-peritoneal methods. The difficult subject of pelvic inflammation is ably treated by Dr. Cullingworth, and the latest views on the question of operation are chronicled. He believes in less radical methods than many Continental surgeons, and concludes that "the indiscriminate removal of the uterus in all cases of operation for inflammatory disease of the appendages is unjustifiable." The article on ovariotomy by the late Mr. Greig Smith follows the lines laid down in his *Abdominal Surgery*, and has a melancholy local interest, as it is amongst the last writings of our eminent townsman.

In mentioning those few points which more particularly interested us in the perusal of this work, we wish it to be clearly understood that we are not making any invidious distinctions as to the value of the many excellent articles which form a volume that reflects much credit both on co-editors and contributors. The index might be extended with advantage. We looked in vain for hæmatometra and hæmatokolpos, and although we found them casually mentioned in the text (p. 345), we failed to find an account of these conditions. A wrong reference is given for kraurosis vulvæ, which should be p. 384, and not p. 584 as stated. "Odium" albicans occurs on p. 385.

